# A Latent Variable Approach for Meta-Analysis of Gene Expression Data from Multiple Microarray Experiments

**DOI:** 10.1186/1471-2105-8-364

**Published:** 2007-09-27

**Authors:** Hyungwon Choi, Ronglai Shen, Arul M Chinnaiyan, Debashis Ghosh

**Affiliations:** 1 Department of Biostatistics, University of Michigan, Ann Arbor, MI, USA; 2 Departments of Pathology and Urology, University of Michigan, Ann Arbor, MI, USA; 3 Department of Statistics and Huck Institute for Life Sciences, Penn State University, University Park, PA, USA

## Abstract

**Background:**

With the explosion in data generated using microarray technology by different investigators working on similar experiments, it is of interest to combine results across multiple studies.

**Results:**

In this article, we describe a general probabilistic framework for combining high-throughput genomic data from several related microarray experiments using mixture models. A key feature of the model is the use of latent variables that represent quantities that can be combined across diverse platforms. We consider two methods for estimation of an index termed the probability of expression (POE). The first, reported in previous work by the authors, involves Markov Chain Monte Carlo (MCMC) techniques. The second method is a faster algorithm based on the expectation-maximization (EM) algorithm. The methods are illustrated with application to a meta-analysis of datasets for metastatic cancer.

**Conclusion:**

The statistical methods described in the paper are available as an R package, metaArray 1.8.1, which is at Bioconductor, whose URL is .

## Background

With the increasing availability of published microarray data sets, there is a tremendous need for developing approaches to validate and integrate results across multiple studies. One major issue to deal with in the meta-analysis of DNA microarrays is the lack of a single standard experimental platform for data generation. The dominant technologies so far have been two-color microarrays and oligonucleotide (e.g., Affymetrix GeneChip) arrays. Because these technologies measure fundamentally differing genetic materials designed to represent identical targets, many properties of expression measurements may vary across platforms including scale of measurements, sensitivity in detecting fold changes, control of cross-hybridization, and so forth. The heterogeneity in array design poses a great challenge for cross-platform comparisons and integration of results across independent microarray studies. The general area of combining data across multiple studies is referred to as meta-analysis [1,2].

Many approaches have been proposed for meta-analysis of microarray data. Rhodes *et al*. [3] combined evidence of differential expression using a summary statistic involving the p-values from comparing cancer versus normal samples across multiple gene profiling studies and adjusted for multiple testing using q-values [4]. Choi *et al*. [5] proposed a Bayesian model for the effect size for genes from multiple microarray experiments. In a more recent study [6], data from one study were used to generate a prior distribution of the differences in logarithm of gene expression between diseased and normal groups, whose distribution was then updated using other microarray studies. These methods all model the effect size [1], or a transformation thereof, across multiple studies.

Recently, we proposed a Bayesian mixture model-based transformation of DNA microarray data based on a proposal of Parmigiani *et al*. [7] and applied it to develop a signature of breast cancer recurrence across multiple microarray experiments from different platforms [8]. The scale which was combined across studies is termed probability of expression (POE). The focus of Shen *et al*. [8] was on the breast cancer application; here, we wish to examine the technical aspects of the modelling used there. Based on the probabilistic model that underlies the POE methodology, one can exploit the notion of using latent variables for combining genomic data from multiple genomic studies. This is a very important idea that can have more general applications than that considered by Parmigiani *et al*. [7]. In **Methods**, we describe the data structure and define two general probabilistic models for quantities that are combinable across studies. The first is the model used in [8]; we present it here for completeness. The second is a two-component mixture model that can be fit using an expectation-maximization algorithm. We also relate the latent variables to recent statistical methodologies for differential expression as well as false discovery rate [4,9]. We then illustrate the proposed methods with an application to a meta-analysis of data comparing metastatic to localized cancer across multiple microarray studies in the **Results **section.

## Results

### Metastatic Cancer Study

We now discuss the application of the proposed methodology to a study looking at metastatic cancer. Based on the availability of expression data for metastatic samples and clinical information regarding the distinction of primary and metastatic tumors, we selected three studies from publicly available data sources [10-12]. These three studies were selected based on two criteria: 1) both localized and metastatic samples are profiled, and 2) a reasonable number of common genes appear across datasets. It should be noted that generally only a small number of metastatic samples are profiled, which was the case in all three datasets. Throughout the article, the terms primary and localized will be used interchangeably.

The goal of this meta-analysis is to identify the set of genes that best distinguishes metastatic tumors from primary tumors in human cancer tissue samples across distinct organ sites. The method mentioned in the previous section is applied to the three training sets to transform the data to POE using both the EM and MCMC algorithms, and an optimal signature based on leave-one-out cross-validation logistic regression framework is obtained. The method will be compared to a few alternative meta-analytic approach ([5,13]) in terms of the selected gene signatures and the clustering of primary and metastatic tumors based on them. Although the validation of methodology is challenging, we used our gene signature to predict metastasis-free survival time in the breast cancer study proposed by van't Veer *et al*. [14] as a possible validation. The hypothesis presumed here is that the profile for distinguishing metastatic from nonmetastatic tumors can be used to predict aggressive cancer prognosis.

### Data Description

Chen *et al*. [12] mainly focus on characterizing the global gene expression patterns that distinguishes hepatocellular carcinoma (HCC) from non-HCC samples using cDNA microarrays. Our sample size numbers (see Table 1) are different from theirs because we have excluded non-tumor samples as well as repeat samples on the same patient. Removing these samples leaves us with 69 unique primary tumors and 9 liver tumors which have metastasized.

**Table 1:** Description of data used in meta-analysis

Data Source	Array Type	Organ Site	Sample	# Metastatic	# Primary
Chen *et al*.	cDNA	Liver	75	9	69
Garber *et al*.	cDNA	Lung	33	6	27
Latulippe *et al*.	Affy U95 Human	Prostate	32	9	23

Garber *et al*. [11] describe the diversity of gene expression patterns in squamous cell carcinomas (SCC), large cell lung carcinomas (LCLC), small cell lung carcinomas (SCLC), and adenocarcinoma (AC) using cDNA microarrays. These four subtypes of lung cancer are often detected in epithelial cells that line different sections of airways in the lung, and their treatment options differ by these types due to the pathological distinction among them. We first selected all 6 unique metastatic tumors and removed their paired samples profiled at primary stage. Identifying and removing duplicate samples was performed the same way as for the Chen *et al*. data. The subset of patients included in our meta-analysis were 27 primary adenocarcinoma samples and 6 samples with lymph node metastases.

Finally, the Latulippe *et al*. [10] study identifies genes that differentiates primary and metastatic cancers in the prostate. Using Affymetrix oligonucleotide array U95 human gene arrays, they reported gene expression profiles of nearly 25,000 genes/ESTs. All samples were included in our meta-analysis. The details for the three studies are summarized in Table 1.

An important aspect of this collection of data is that the organ sites are different. We are postulating a hypothesis that there is a common profile separating localized tumors from metastatic tumors across the three sites. Similar evidence for this type of hypothesis has been suggested before [15]. The microarray platform differs by studies, so we mapped clone/probeset IDs to Unigene cluster IDs (UGIDs) of its most recent build through SOURCE [16]. UGIDs are constantly updated. Because our initial mapping was done in the year 2004, we translated these UGIDs to the June 2006 build (No. 191) in the NCBI database. The genes we report here and their annotation in the remainder of the paper is consistent with all annotations associated with the most up-to-date Unigene clusters. When multiple clones are mapped to the same UGID, we averaged the expression over the clones within each sample. Such a mapping produced 1633 common UGIDs.

### POE

Before combining 140 samples from different sources into a single dataset, we transformed the raw data to POE from each study by normalizing the distribution of expression values in metastatic samples to that of localized samples. Note that the localized or primary tumors represent the baseline group, since our goal is to select gene signature that distinguishes metastatic tumors from localized tumors, for which many conflicting hypotheses have been postulated. The output of POE from each study was then combined to form a single expression dataset with 1633 genes and 140 samples.

In the following, the POE data transformations by the EM and MCMC algorithms will be analyzed in parallel for the sake of comparison. All primary tumors are color-coded in red and metastatic tumors in green. In terms of computational speed, estimation of POE based on the EM algorithm takes less than a minute for 1633 genes per dataset, while that using MCMC takes about 50 minutes for 2000 iterations and 4 periodic skips in the sampler. As the numbers of genes and samples grow, this difference will be substantial. For example, it usually takes 4 hours to fit POE for a dataset with 10,000 genes using full Bayesian modelling as opposed to 3 minutes for the maximum likelihood approach using the EM algorithm. The reason for the computational difference is that the EM algorithm is fit to one gene at a time, while the MCMC algorithm involves fitting to expression measurements for all genes simultaneously.

**Figure 1:**
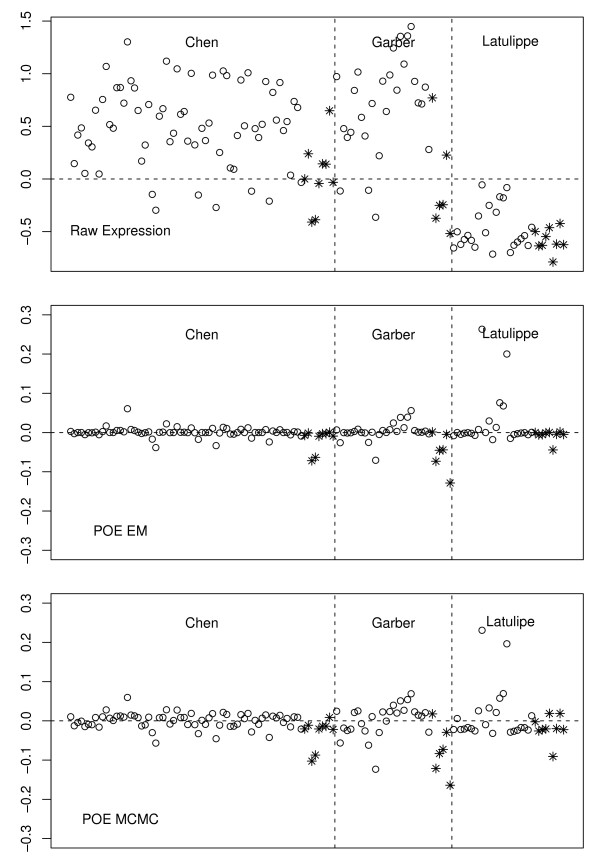
**Expression of TGFB1**. Transforming growth factor beta 1 (TGFB1) gene expression on raw (upper), POE EM (middle) and POE MCMC (lower) scales. This gene is uniformly underexpressed in metastatic samples. Open circles indicate primary tumor samples, and stars indicate metastatic samples.

**Figure 2:**
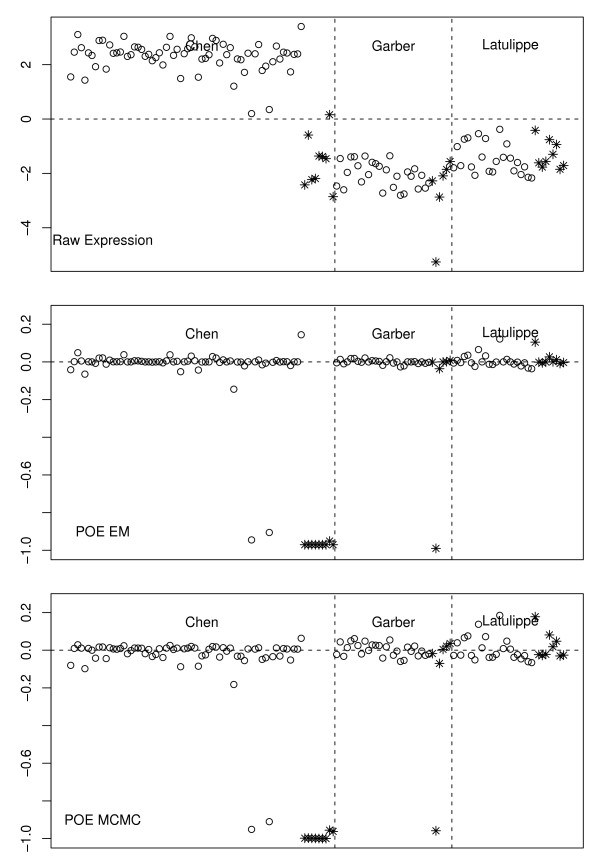
**Expression of F2**. F2 coagulation factor II (F2) gene expression on raw (upper), POE EM (middle) and POE MCMC (lower) scales. This gene is underexpressed primarily in metastatic samples of the Chen liver study. Open circles indicate primary tumor samples, and stars indicate metastatic samples.

Figures 1 and 2 show the POE transformation for two genes using both the EM and MCMC algorithms. In both plots, the top panel shows the expression levels on the raw scale, followed by those on the POE scale from the EM and MCMC algorithms, respectively. The gene in Figure 1 is TGFB1 (UGID Hs.155218), which controls proliferation and differentiation in many cell types. The gene in Figure 2 is F2 (UGID Hs.410092), coagulation factor II, whose mutation leads to various forms of thrombosis and which is often expressed in liver tissues.

Although both genes are in the signature obtained by our methods, they clearly represent different types of genes. Based on Figure 1, F2 is under-expressed in the metastatic liver samples of Chen *et al*., weakly under expressed in the lung samples of Garber *et al*., and not differentially expressed in the Latulippe *et al*. data. It was found significant only in the liver study among the three studies we considered here. On the other hand, TGFB1 is a gene whose expression is uniformly under expressed in metastatic samples across all three studies.

This observation on the two types of expression pattern on POE scale suggests that our signature will contain both types of genes. As will be shown later, a conventional meta-analytic approach that combines strength of differential expression across studies on the raw scale tends to select genes that behave similarly to TGFB1, whereas our method picks up both types of genes. Unless genes with expression patterns similar to F2 dominate the entire signature, the gene set from our method tends not to be influenced by a single study.

### Signature Selection

As we proposed POE transformations using two different implementations, we will refer to the signatures from the data transformed by the EM and MCMC algorithms as the POE EM signature and the POE MCMC signature, respectively.

To obtain a gene signature that distinguishes metastatic samples from localized samples, we calculated risk indices for all samples. What we call a risk index is described in the Methods section. A logistic regression is fitted for each gene with one sample held out at a time. The response variable is metastasis status (1 = metastatic, 0 = localized). For all genes we iterated the same procedure holding each sample out while recording coefficients *β *and *p*-values. Following the risk index approach for classification expalined in Methods section, we calculated risk indices for all 140 subjects at various sizes of the gene signature. The optimal signature size *p *was then determined based on classification performance.

**Figure 3:**
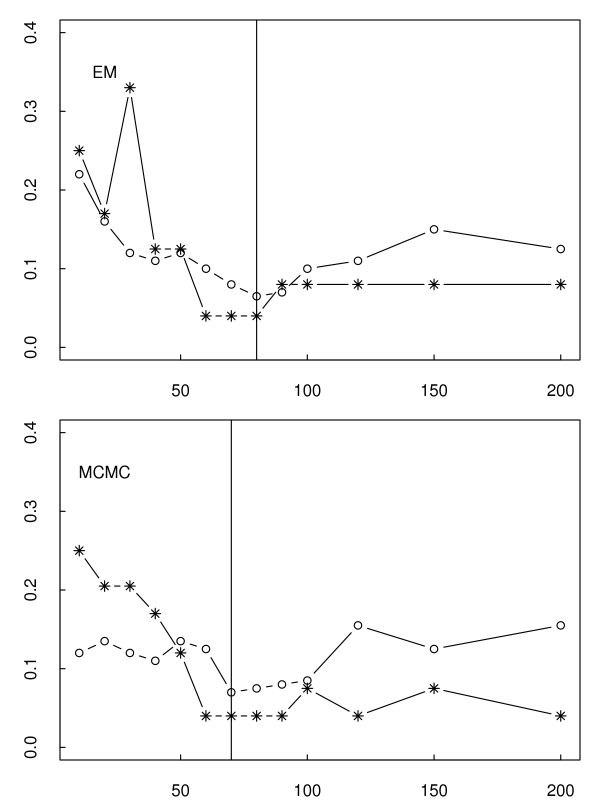
**Misclassification Error**. Misclassification error rates in metastatic (starred line) and primary tumors (open circled line). The upper panel is the error rates from the data transformed by the EM algorithm, and the lower panel is that from the data transformed by the MCMC algorithm.

**Figure 4:**
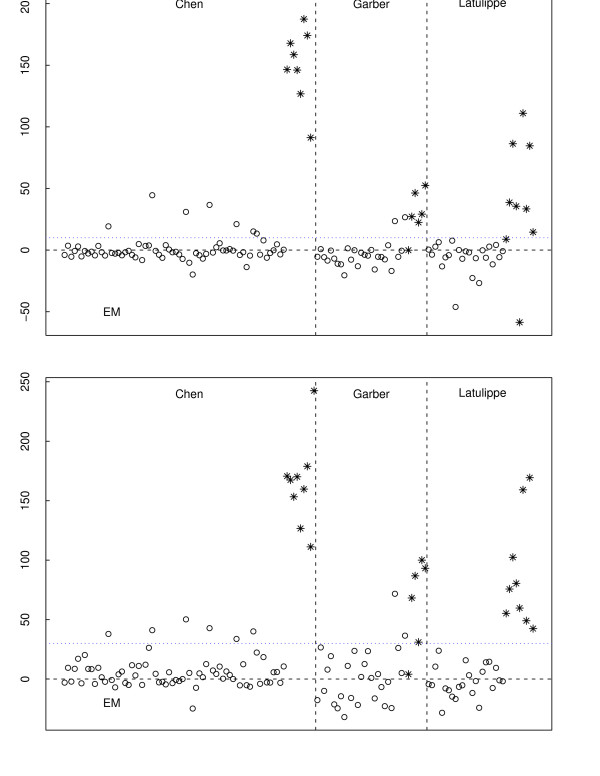
**Risk index**. Derived risk indices from the data transformed by the EM and MCMC algorithms. Primary and metastatic tumors are represented by open circles and stars respectively. The y-axis is risk index.

For classification purposes, we predicted the subjects with positive risk index to be metastatic and those with negative risk index to be localized cancer. Using Figure 3, we took the optimal size to be 80 for the POE EM signature as the error rates in metastatic and primary tumor samples collectively reach a minimum and do not decrease further as more genes are added beyond 80. A similar criterion was applied to obtain a 70-gene POE MCMC signature. A plot of the risk indices and the optimal cutpoint is given in Figure 4. The POE EM and POE MCMC signatures share 52 common UGIDs.

### Comparison and Validation

We performed other analyses for the sake of comparison. First, we compared the classification performance of the signatures found using meta-analyses with that in which the classifiers were constructed on one dataset only and tested on the other two datasets. The performance is summarized in Table 2. While such individual study-specific signatures tended to perform well on the training dataset, their performance did not generalize well to other datasets. The consistently poor performance of all signatures on the Garber dataset, including its own signature, suggests that this dataset might have poorer reliability than the others within the common subset of 1633 genes used in this analysis.

**Table 2:** Classification error rates

	Chen	Garber	Latulippe
Chen (50)	0	27	21
Garber (25)	18	12	18
Latulippe (30)	17	21	0

POE EM (80)	2	6	0
POE MCMC (80)	1	6	0

Effect Size (80)	1	12	0
Conlon *et al*. (80)	7	27	0

Table entries are misclassification error rates in percentage points using classifier from study on row to predict that listed in the column. Number in parentheses in the signature column refers to number of genes at which classification accuracy was optimized. E3ect Size refers to method of [5].

We also compared our methods with two meta-analysis techniques developed in [5] and [13]. The former performs Bayesian inference on the classical Hedges-Olkin pooled effect sizes for each gene from multiple studies, and the latter uses Bayesian hierarchical model to pool datasets across studies through group-specific mean and variance parameterization and selects gene signature based on their Bayesian estimate of FDR.

**Figure 5:**
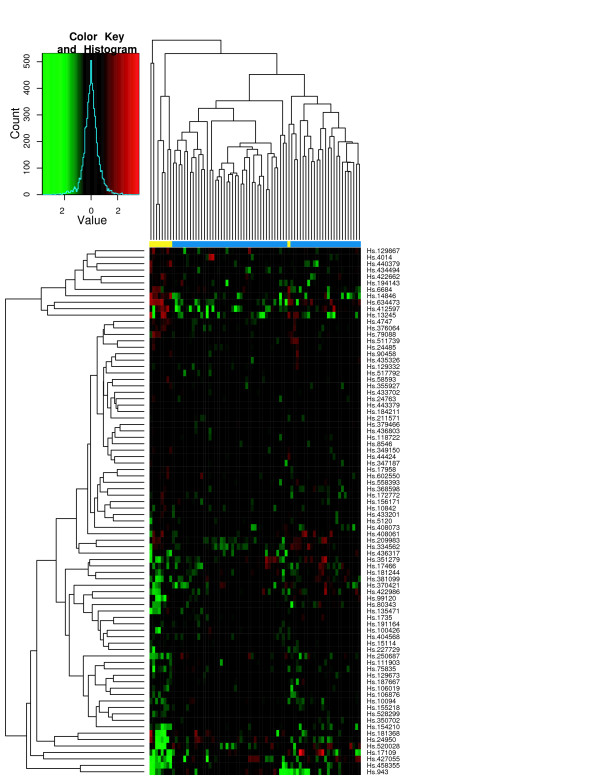
**POE and Conlon *et al*. comparison**. Concordance of the gene specific probability of differential expression between the POE (EM) and the method in [13].

First, since the method of [5] pools the differential expression statistics from a collection of raw-scale data, there is no analogue of a risk index-based classification method available using their signatures. Instead, we first obtained a signature of size 80 based on univariate gene selection. Here the choice of size 80 in all signatures was chosen to provide a fair comparison of class prediction power with POE signatures. This corresponds to controlling the FDR at 0.02 in the method by [5]. We call this the effect size (ES) signature. We also fitted the hierarchical model from [13] using WinBUGS software [17]. We used the prior specification reflecting vague prior information as in the original paper. The fitted model was obtained from a simulation of 12,000 iterations with the initial 2,000 iterations used for burn-in. The estimated probabilities of differential expression were surprisingly low, with the highest probability 0.003. This implies that the 80 gene signature has FDR 99%. For the sake of comparison, we also took the 80 gene to assess its class prediction ability. We call this Conlon signature. Since both POE and the latter method report the probability of differential expression of individual genes, we examined the concordance between the two sets of probabilities. Figure 5 shows the probability in Conlon *et al*. plotted against that in POE EM. The ES signature shared 15 UGIDs in common with the POE EM signature and 18 genes with the POE MCMC signature only, which suggests that the two signatures will have different characteristics.

Meanwhile, the Conlon signature had an overlap of two genes with the ES signature and one gene with the POE EM and MCMC signatures. The poor overlap of Conlon signature with others is consistent with the high Bayesian FDR estimated above.

**Figure 6:**
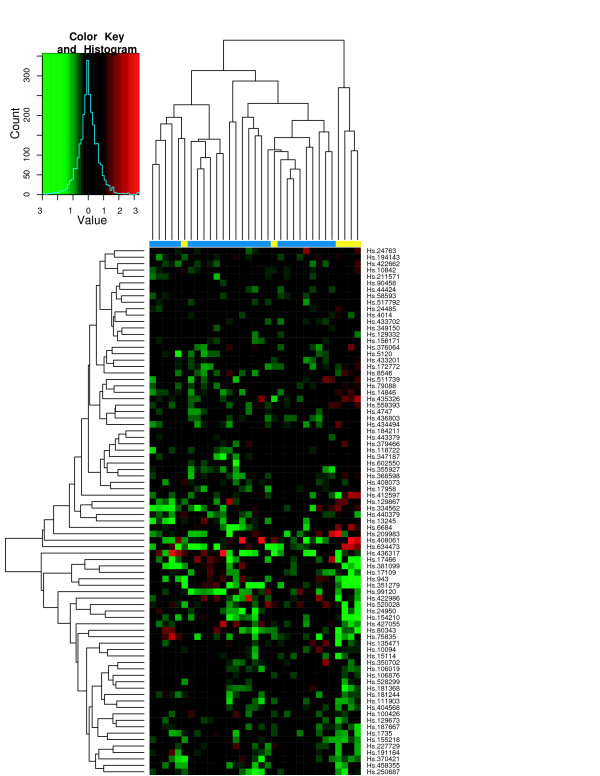
**Chen *et al*. data**. Hierarchical clustering of tumors in Chen *et al*. data using the effect size signature. The expression here is on the raw scale. The color strip in blue and yellow below the heatmap indicates primary and metastatic tumors. Blue indicates primary tumors and Yellow indicates metastatic tumors.

**Figure 7:**
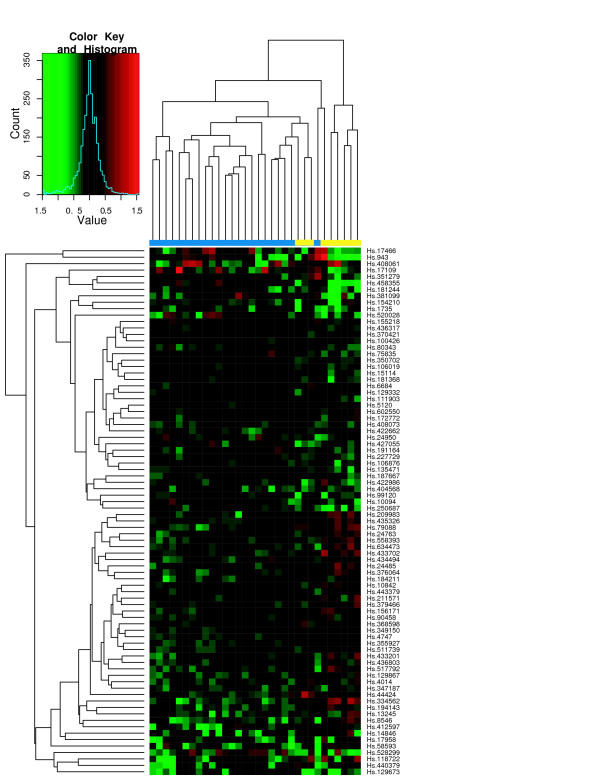
**Garber *et al*. data**. Hierarchical clustering of tumors in Garber *et al*. using the ES signature. The expression here is on the raw scale. The color strip in blue and yellow below the heatmap indicates primary and metastatic tumors, respectively

**Figure 8:**
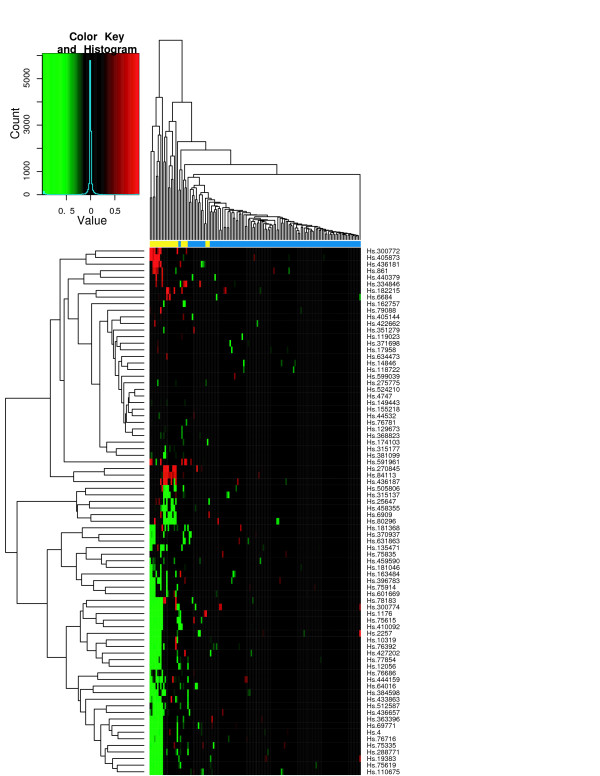
**Latulippe *et al*. data**. Hierarchical clustering of tumors in Latulippe *et al*. using the ES signature. The expression here is on the raw scale. The color strip in blue and yellow below the heatmap indicate primary and metastatic tumors, respectively.

**Figure 9:**
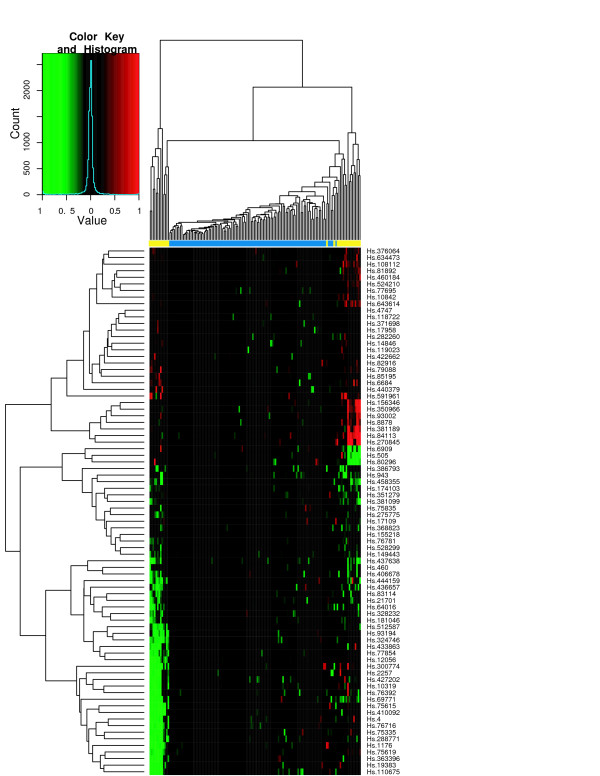
**POE EM for all three datasets**. Hierarchical clustering of tumors of all three studies using the POE EM signature. The expression is on the POE scale.

**Figure 10:**
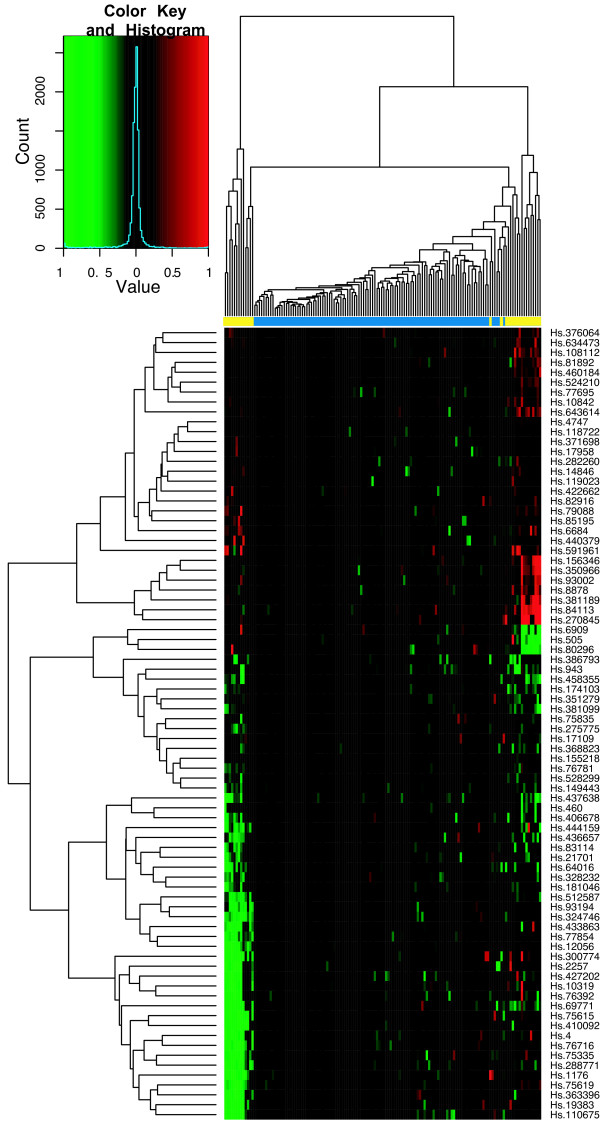
**POE MCMC for all three datasets**. Hierarchical clustering of tumors of all three studies using the POE MCMC signature. The expression is on the POE scale.

To assess the classification performance, we performed hierarchical clustering of tissue samples from the individual studies using the ES signature. Figures 6 through 8 show the heatmaps of the ES signature in individual studies with clustering tree. These were drawn separately because the raw scale data cannot be directly combined as in POE. Figures 9, 10 are the heatmaps of the POE EM and MCMC signatures. To highlight the sample labels in each plot, a yellow/blue color strip was added to the top of the dendrograms through Figures 6, 7, 8, 9, 10, which should be viewed along with the breakdown of the clustering tree. For all plots, we used average linkage clustering with the distance metric defined using the Euclidean metric. This was also done for the Conlon signature [see Additional Files 1, 2, 3]. We found that the clustering performance of this signature was similar to that in the ES signature as well, with most of the errors committed in Garber lung study. The overall classification performance across all signatures is provided in Table 2. Based on the classification table, we see that the proposed methods (EM and MCMC) greatly outperform the Conlon signature, while they also are superior to the ES method, although this difference is smaller.

We note that clustering with all signatures give fairly accurate results in all three studies. In the ES signature, only a few metastatic samples are grouped together with two other primary tumors for the Chen liver study (Figure 6). Two metastatic samples are situated under the same node with primary tumors in Garber lung study (Figure 7), Finally, one primary and another three metastatic samples are in the opposite clusters in Latulippe prostate study (Figure 8). Overall, the clustering can differentiate metastatic tumors from primary tumors, although some metastatic tumors were grouped with primary tumors. The Conlon signature had no classification error in Latulippe prostate study, but essentially there was no tight clustering in Garber lung study at all, although 4 out of 6 metastatic samples were clustered together in a local tree.

The POE EM and MCMC signatures give comparably good clusterings of the two types of tumors across all studies. In Figures 9 and 10, all metastatic tumors except for two samples from the Garber lung study are grouped together, and some primary tumors from the Chen liver study are separated from other primary tumors. Furthermore, the lengths of the edges to the leaf nodes in the dendrogram are shorter than that in the ES signature, which suggests that the clustering of primary tumors is tighter than that using the ES signature. This is a consequence of normalizing the expression level of metastatic tumors to the distribution of primary tumors by utilizing phenotypic information in the estimation of POE. The heatmaps visually demonstrate the difference between the ES signatures and the POE signatures. We next used NIH DAVID [18] to determine if there were functional groups enriched for in our gene expression signatures. In terms of gene annotation, the POE EM and MCMC signatures share many common functional categories because they have many UGIDs in common such as response to stress, immune response, endopeptidase and enzyme inhibitor activity, cell organization and biogenesis, and regulation of cell cycle. The class of functions common to the POE and ES signatures is cell cycle processes. GO terms such as antigen processing, endogenous antigen via MHC class I, DNA repair, many metabolism and transport activities appear in the ES signatures only. Also, a literature search has suggested the association of POE signature genes and their corresponding GO terms with tumor invasion and metastasis in various cancer types. For example, ALDH1A1 (stress response) and MAPK3 (cell proliferation) are targets of the HGF/MET signaling pathway which has been associated with tumor metastasis and poor prognosis in human hepatocellular carcinomas [19]. In another example, overexpression of PFN2 (Regulation of actin cytoskeleton) and UBS (stress response) has been associated with lymph node metastasis of gastric cancer [20] and colon cancer [21] respectively. These observations indicate that the POE signatures lead to relevant findings toward understanding the potential mechanism of differentiation of metastatic tumors from primary tumors.

Finally, an additional validation of the method was attempted to see if the resulting gene expression signature can discriminate lethal from nonlethal cancers in an early detected population of cancers. Note that the signature selection was primarily oriented toward the distinction of metastatic tumors from primary tumors. Thus validation here is based on the conjecture that many metastatic tumors are highly likely to initiate lethal condition. We addressed this issue by using the data from the van't Veer *et al*. [14] study. Their study profiled 98 primary breast cancer samples in Hu25K inkjet arrays. Among these patients, 34 patients developed distant metastases within 5 years, 44 patients continued to be disease-free after a period of at least 5 years. Other 20 patients either had BRCA1 germline mutations or were BRCA2 carriers; we excluded these samples from the analysis.

The study was based on a large inkjet microarray profiling over 25,000 probes. About two-thirds of 1633 genes used in the three cancer studies appear in the Van't Veer *et al*. data. Based on the classifier trained from the three cancer datasets described above, we mapped the genes from the signatures to those in the van't Veer *et al*. data. We generated risk indices for subjects in van't Veer *et al*. Specifically, we first transformed the van't Veer *et al*. data to the POE scale using both the EM and MCMC algorithms without using the phenotypic information to prevent overfitting. Note that we did not consider the effect size and Conlon signatures here. Then we calculate the log odds ratio for each patient using the coefficients trained from training data and the newly generated POE data. Note that the estimated regression coefficients for the risk score came from the training set. As expected, the derived risk indices using the data from the EM and MCMC algorithms are highly correlated (Pearson correlation 0.83).

A proportional hazards model [22] relating metastasis-free survival to the risk index, adjusting for covariates, was fit to the data. Tables 3 and 4 shows the results. In both analyses using data from the EM and MCMC algorithms concur in that the derived risk indices are strong predictor of metastasis-free survival times. This association remains strong even after adjusting for estrogen receptor status and age. Since we are interested in risk prediction, we calculated the C-index [23] to see if the gene expression signature adds discriminatory information relative to estrogen status and age. For the model with just age and estrogen status, the C-index is 0.714. For the EM-based POE signature, the C-index with all three variables (Multivariate model in Table 3) is 0.722. For the MCMC-based POE signature, the C-index with all three variables (Multivariate model in Table 4) is 0.748.

**Table 3:** Results of EM POE-based survival analysis

Analysis	Variable	Coef	*p*-value
Univariate	Risk Index	0.015	0.005

Multivariate	Risk Index	0.010	0.049
	Estrogen Receptor Pos	-0.697	0.058
	Age	-0.059	0.021

Cox proportional hazards model fitted to time to distant metastasis in lymph node from study by [14]. Risk indices were derived based on POE data transformed by the EM algorithm.

**Table 4:** Results of MCMC POE-based survival analysis

Analysis	Variable	Coef	*p*-value
Univariate	Risk Index	0.036	< 0.001

Multivariate	Risk Index	0.027	0.008
	Estrogen Receptor Pos	-0.580	0.120
	Age	-0.056	0.030

Cox proportional hazards model fitted to time distant metastasis in lymph node from [14]. Risk indices were derived based on the POE data transformed by the MCMC algorithm.

## Discussion

Ideally, we wish to use all common genes from the available studies for meta-analysis. However, one issue that has been debated recently is that of reproducibility of genes across studies [24,25]. A technique that has proven to be useful as a filtering device to enhance comparability across arrays of different platforms is the integrative correlation coefficient or correlation of correlation coefficients [24,25]. The idea underlying this method is that while raw expression values vary from study to study, the intergene correlations do not vary as much. The intergene correlations are calculated across all samples; this yields *a N × N *matrix for each study. The row-wise averages are taken for each study and then calculated, and the correlations of these averages between these studies is then calculated. Thus, one would consider combining genes that have similar intergene correlations across the studies. For normally distributed data, the sample correlation is independent of the sample mean. Thus, genes selected based on an integrative correlation filter need not necessarily be highly expressed genes. We could perform this as a filtering step before applying the proposed meta-analysis methodologies; we did not do so here. The drawback to such a measure is that the filtering of genes might reduce the chance of finding subtypes in the datasets because the genes that define such subtypes may be excluded based on the integrative correlation coefficient.

One limitation of our methodology is that it is still subject to the usual meta-analysis assumption that the transformed expression measurements (the POE values) are directly combinable across studies. If in fact we are trying to combine measurements for fundamental different quantities across multiple studies, then this in fact renders the meta-analysis invalid. However, for that situation most meta-analytical approaches are invalid, and one would need more sophisticated modelling assumptions.

A related issue to this is that of heterogeneity. The results of the analysis here should be interpreted with some caution in that we are comparing metastatic versus nonmetastatic tumors across a variety of tissue types. We made the assumption that the differences between the two types of tumors are the same across the three studies. If this is not true, then it might be quite possible that what we are detecting are in fact tissue-specific differences between metastatic and non-metastatic tissues. It is of interest to develop methods for assessing heterogeneity in meta-analyses of genomic data so that they may be applied before using the proposed methodology in the paper.

## Conclusion

With the proliferation of genomic datasets from related studies by different scientific groups, an important method for increasing power will be to combine results across the different studies. In this article, we have proposed a model-based approach to doing this. Being able to integrate and interpret multiple genomic datasets will be an important enterprise for data analysts working in bioinformatics to address in the future.

A question orthogonal to that looked at in this paper but of equal scientific importance is that of identifying genes whose expression is correlated across a subset of the samples. This is referred to as molecular subtype analysis and was in fact one motiviation of the POE algorithm proposed by Parmigiani et al. (2002). However, finding such gene signatures would require the use of completely different statistical methods than those proposed here and is beyond the scope of the current paper.

Several important issues to consider when integrating microarray studies include use of different gene expression measurement scales, varying analytical power and reliability of the results for individual studies. To address these issues in a meta-analysis framework, we proposed a two-stage mixture modeling strategy. The goal of the mixture model-based transformation is to transform the preprocessed data to the probability scale, which are then integrated across datasets. In particular, the signed probability of differential expression *p*^*d *^is easily interpretable and is platform-independent. The Normal-Uniform mixture distribution under a Bayesian hierarchical model setting has several desirable properties. We have also proposed an alternative model based on a two-component mixture and estimation using the EM algorithm. We briefly compare the MCMC and EM algorithms. The advantage of the former method is that it pools information across all genes, while the latger approach does not. However, the EM algorithm is computationally much faster than the MCMC scheme. In our example, we find that there is substantial overlap between the two approaches for the metastasis data considered here. However, we also expect that for cancer studies, the EM algorithm would fare better with larger phenotypic differences (e.g., non-cancer versus cancer tissue), while the MCMC approach would be of use when the phenotypic differences in samples are subtle (e.g., Gleason score ≤ 7 versus > 7 in prostate cancer).

Combining samples on the probability scale mitigates the influence of potential artifacts from a single study. The effect is reflected on two counts. One, integrated sample cohorts improve the reliability of the findings by guarding against false positive results from a single study. Two, it increases the statistical power to detect small consistent effects that can be otherwise masked by inadequacy of the sample size of an individual data set. By implementing this modeling approach, we were able to combine information from three microarray studies to build an inter-study validated signature for discriminating metastatic cancer from non-metastatic cancer.

The statistical methods described in the paper are available as an R package, metaArray, which is available through the Bioconductor project at [26].

## Methods

### Data Structures and Probabilistic Models

Let $x_{ij}^{k}$ denote the gene expression measurement for gene *i *from sample. *j *in study *k*, transformed using the base two logarithm, *i *= 1, ..., *N*, *j *= 1, ..., *M*_*k*_, *k *= 1, ..., *K*. Note that we assume that there are *N *common genes in all *K *studies, but the number of arrays in studies may vary. We also assume that preprocessing has been done, either by a lowess normalization for two-channel microarray data [27] or a robust multichip average analysis for Affymetrix data [28]. Then the available data can be denoted by ${\left\{X^{k}\right\}}_{k=1}^{K}$, where *X*^*k *^is a *N *× *M*_*k *_matrix whose (*i*, *j*)th entry is $x_{ij}^{k}$. Note that the value and interpretation of $x_{ij}^{k}$ is inherently different across array platforms and is not necessarily comparable if they are measured from independent studies.

Corresponding to $x_{ij}^{k}$, let $e_{ij}^{k}$ be a variable that takes one of three values {1, 0, -1}, indicating over-, baseline- or under- expression respectively for gene *i *in sample *j *for the study *k*. If $e_{ij}^{k}$ were known, then this is a gene-specific quantity that would provide a platform-free scale which could be combined across multiple studies. We approach this problem by treating $e_{ij}^{k}$ as a latent variable that is inferred from the data using a mixture model. We now present two probabilistic specifications for making inference about $e_{ij}^{k}$. The first was presented in [8]; we describe it here for the sake of completeness. The second assumes $e_{ij}^{k}$, take two values of 0 and 1 and involves fitting a two-component mixture model using the EM algorithm. Both specifications aim to map the original expression values to POE values within a study. We then can combine the POE values across the multiple studies. In the simplest case, a direct group comparison can be made by calculating *t*-statistics or applying significance analysis of microarrays [29] to the combined data. In the remainder of this section, we describe the two approaches for obtaining POE expression values. From here on, we suppress the study indicator *k *throughout this section because estimation is performed within each study separately, with the only exception being the use of *M*_*k *_to denote the number of samples in study *k*.

### Bayesian Model-based Approach and Algorithm

This approach was first explored for a single study setting in [7] and used in the meta-analysis setting in [8]. We present it here for the sake of completeness. The estimation of the POE values involves borrowing information across all genes.

First, the model specification is described. Following the approach of [7], we assume that the expression *x*_*ij *_of gene *i *in sample *j *is a realization of the following mixture model:

$$\begin{array}{cc} x_{ij}|\mu_{i},\alpha_{j},\kappa_{i}^{+},\kappa_{i}^{-},\sigma_{i}^{2}\overset{ind}{\underset{}{\sim }} & \pi_{i}^{+}U(\mu_{i}+\alpha_{j},\mu_{i}+\alpha_{j}+\kappa_{i}^{+})\\ & +(1-\pi_{i}^{+}-\pi_{i}^{-})N(\mu_{i}+\alpha_{j},\sigma_{i}^{2})\\ & +\pi_{i}^{-}U(\mu_{i}+\alpha_{j}-\kappa_{i}^{-},\mu_{i}+\alpha_{j}) \end{array}$$

where *μ*_*i *_is the gene-specific effect of gene *i*, *α*_*j *_is the sample-specific effect in sample *j*. For the purposes of identifiability, sample effects ${\left\{\alpha_{i}\right\}}_{i=1}^{M_{k}}$ are constrained to sum to zero. The parameters $\kappa_{i}^{+}$ and $\kappa_{i}^{-}$ provide limits to the uniform distribution in the mixture of gene *i*, and are set to be at least either 3*σ*_*i *_away from the mean of normal distribution or farther away than the most outlying expression. The parameters $\pi_{i}^{+}$ ≡ *P*(*e*_*ij *_= 1) and $\pi_{i}^{-}$ ≡ *P*(*e*_*ij *_= -1) are the multinomial probabilities for the latent variable $e_{ij}^{k}$. Conceptually, we can think of gene expression for the *i*-th gene arising from three types of genes in model (1). The first component in the model represents the type of genes whose expression levels are overexpressed in the cancer samples relative to the normal samples. The second corresponds to genes that do not change between cancer and normal samples, and the third is for genes that are underexpressed in cancer samples relative to normal.

Let $p_{ij}^{+}$ ≡ *P*(*e*_*ij *_= 1|*x*_*ij*_) and $p_{ij}^{-}$ ≡ *P *(*e*_*ij *_= -1|*x*_*ij*_) be the conditional probabilities of over and underexpression for gene *i *in sample *j *given the microarray measurements. By Bayes' rule,

$$p_{ij}^{+}=\frac{\pi_{i}^{+}f_{1i}(x_{ij})}{\pi_{i}^{+}f_{1i}(x_{ij})+\pi_{i}^{-}f_{-1i}(x_{ij})+(1-\pi_{i}^{+}-\pi_{i}^{-})f_{0i}(x_{ij})}$$

and

$$p_{ij}^{-}=\frac{\pi_{i}^{-}f_{-1i}(x_{ij})}{\pi_{i}^{+}f_{1i}(x_{ij})+\pi_{i}^{-}f_{-1i}(x_{ij})+(1-\pi_{i}^{+}-\pi_{i}^{-})f_{0i}(x_{ij})}$$

where *f*_0*i *_is the normal density function, and *f*_1*i*_, *f*_-1*i *_are the corresponding uniform densities for the differential expression categories for the gene *i *in each study. In the numerator of (2), *f*_1*i *_= 1/$\kappa_{i}^{+}$ if *x*_*ij *_∈ [*μ*_*i *_+ *α*_*j*_, *μ*_*i *_+ *α*_*j *_+ $\kappa_{i}^{+}$] and 0 otherwise. In the numerator of (3), *f*_-1*j *_= 1/$\kappa_{i}^{-}$ if *x*_*ij *_∈ [-$\kappa_{i}^{-}$ + *μ*_*i *_+ *α*_*j*_, *μ*_*i *_+ *α*_*j*_] and 0 otherwise.

Note that the supports of the two uniform distributions are disjoint. As a result, the probabilities of differential expression are mutually exclusive with the following forms:

$$(p^{+},p^{-})=\left(\frac{\pi^{+}/\kappa^{+}}{\pi^{+}/\kappa^{+}+(1-\pi^{+}-\pi^{-})f_{0}},0\right)$$

$$(p^{+},p^{-})=\left(0,\frac{\pi^{-}/\kappa^{-}}{\pi^{-}/\kappa^{-}+(1-\pi^{+}-\pi^{-})f_{0}}\right).$$

We then construct the following expression measurement: $p_{ij}^{d}=p_{ij}^{+}-p_{ij}^{-}$, ranging from -1 to 1. This is the probability of expression (POE); it can be interpreted as the signed conditional probability of differential expression of gene *i *in sample *j *in an individual study. On first glance, our formula differs from that in [7] in that their POE measure, corresponding to $p_{ij}^{d}$, involves addition, while ours involves subtraction. The two are equivalent, however, since their second probability is constrained to be negative, while $p_{ij}^{-}$ is constrained to be positive.

To sample from the posterior distributions of the parameters, a Gibbs sampling algorithm (with Metropolis-Hastings step for mixture proportion parameters) was then implemented where the gene-specific parameters were repeatedly sampled from the corresponding full conditional distributions [See Additional File 4]. We thus fit the Bayesian algorithm to each microarray dataset separately. Note that there is one normal component distribution to the mixture, while the other two are uniform distributions. The reason we prefer this to a three-component normal mixture model is so that the probabilities of expression are monotonic functions of absolute gene expression. It can be shown that using a three-component normal mixture model, the POE is no longer a monotonic function of gene expression. It is desirable to have the monotonicity property as we would like larger differences in gene expression relative to a baseline group to be associated with greater statistical evidence of differential expression. Note that this is a standard assumption made in the meta-analysis methodologies.

There are several advantages to the mixture model-based transformation. First, the method estimates the posterior distribution for the latent variables *e*_*ij*_, which can then be combined across multiple studies. Second, the transformed values carry meaningful interpretations as signed probabilities of differential expression of a gene in a particular sample. Third, the underlying normal and uniform mixture distributions give equal density in the tails and is effective in reducing the influence of extreme expression values. Finally, the Bayesian hierarchical modeling approach borrows strength across genes, resulting in shrinkage-type estimators for the gene-specific parameters. Consequently, the high-dimensional gene expression data are denoised. However, the algorithm for inferring the posterior distribution of the latent variables is fairly computationally intensive. In the next section, we discuss an alternative mixture model specification that leads to a more computationally efficient algorithm.

### Maximum Likelihood Approach using EM algorithm

Maximum likelihood estimation (MLE) using the EM algorithm leads to greater increases in computational speed for mixture models. Such an approach might be useful since what we are interested in eventually is estimates of POE that we can integrate across studies. There is a difficulty in implementing an EM algorithm for the three-component mixture model we considered in the previous section. Recall the restriction that the uniform components must have the same heights. Since the MLE of end points of uniform distributions are the most outlying observations, we have found in some examples that the EM algorithm with these MLEs provides parameter estimates that are unstable.

As an alternative modelling approach, suppose we take the three values for the latent variables *e*_*ij *_from the previous section (*e*_*ij *_= {-1, 0, 1}) and collapse them into two possible values, *e*_*ij *_= 1 and *e*_*ij *_= 0. Note that for the *i*th gene and *j*th sample, *e*_*ij *_= 1 corresponds to differential expression in either direction, while *e*_*ij *_= 0 represents nondifferential expression. We now consider the following two-component mixture model for the *i*th gene:

$$x_{ij}\overset{ind}{\underset{}{\sim }}\pi_{i}U(a_{i},b_{i})+(1-\pi_{i})N(\mu_{i},\sigma_{i}^{2}),$$

where *μ*_*i *_is the mean of the normal distribution, *π*_*i *_is the mixing proportion and *a*_*i*_, *b*_*i *_are the two end points of Uniform distribution respectively. Conceptually, there are only two populations of genes in model (4). There is a constitutively expressed population common to both tumor and normal samples (the normal component) as well as a differentially expressed part (the uniform component). Such a model has been proposed in the situation for *K *= 1 in [30]. Their interest was in determining differentially expressed genes within one study, while ours is in combining results across multiple studies.

For a fixed *i *(gene), the likelihood to be maximized for each gene is the following:

$$\prod_{j=1}^{n}\left[\frac{\pi_{i}}{b_{i}-a_{i}}+\frac{\left(1-\pi_{i}\right)}{{\left(2\pi \sigma_{i}^{2}\right)}^{1/2}}\exp\left\{-\frac{{\left(x_{ij}-\mu_{i}\right)}^{2}}{\sigma_{i}^{2}}\right\}\right].$$

It is simple to find parameters that maximize the mixture model likelihood using the EM algorithm [31]. Maximization is accomplished by iterating between the following steps. In the *E *step, we calculate

$$e_{ij}^{\left(t+1\right)}=\frac{\frac{\pi_{i}^{\left(t\right)}}{b_{i}-a_{i}}}{\frac{\pi_{i}^{\left(t\right)}}{b_{i}-a_{i}}+\frac{1-\pi_{i}^{\left(t\right)}}{{\left(2\pi^{\left(t\right)}\sigma_{i}^{2^{\left(t\right)}}\right)}^{1/2}}\exp\left\{-\frac{{\left(x_{ij}-\mu_{i}^{\left(t\right)}\right)}^{2}}{\sigma_{i}^{2^{\left(t\right)}}}\right\}}$$

where *t *indexes the iteration of the EM algorithm, and *e*_*ij *_is the unobserved indicator for the gene *i *in sample *j *to have originated from the uniform component of the mixture distribution. In the *M *step, we calculate

$$\begin{array}{c} \pi_{i}^{\left(t+1\right)}=\sum_{j}e_{ij}^{\left(t+1\right)}/n\\ \mu_{i}^{\left(t+1\right)}=\frac{\sum_{j}\left(1-e_{ij}^{\left(t+1\right)}\right)x_{ij}}{\sum_{j}\left(1-e_{ij}^{\left(t+1\right)}\right)}\\ \sigma_{i}^{2_{\left(t+1\right)}}=\frac{\sum_{j}\left(1-e_{ij}^{\left(t+1\right)}\right){\left(x_{ij}-\mu_{i}^{\left(t+1\right)}\right)}^{2}}{\sum_{j}\left(1-e_{ij}^{\left(t+1\right)}\right)}. \end{array}$$

As mentioned in [30], *a*_*i *_and *b*_*i *_are estimated by the minimum and maximum of the *x*_*ij *_for a fixed *j*. We obtain estimates of e_*ij*_, ${\hat{e}}_{ij}$, after the EM algorithm converges. To construct estimates of POE, we take ${\hat{e}}_{ij}$ and make it negative for those samples whose observed expression was to the left of the mean of the baseline distribution of expression for each gene. This places the POE values from this model on the same scale as those from the three-component mixture model. In terms of initial values for the mixture model and in particular *e*_*ij*_, they were set at zero for all points except those that were three standard deviations from the mean expression.

Such an approach is much less computationally challenging compared to the Markov chain Monte Carlo described in the previous section. In particular, the MCMC method requires iterating over all *N *genes, while the EM algorithm is calculated for one gene at a time. Letting *S *and *I *denote the periodic skip in Gibbs sampler and the number of iteration respectively, the computation speed is *O*(*NSI*) for the MCMC algorithm, while it is *O*(*N*) for the EM algorithm. We compare the performances of the two methods on a real dataset in the **Results **section.

Until now the discussion of transforming data into POE values has implicitly assumed that no phenotype information was used in the estimation algorithm. Nonetheless, there is often phenotypic data available in experiments, which provides *a priori *information on the *e*_*ij*_. In the example considered here, the goal of meta-analysis is to compare localized tumors from metastatic tumors. By using phenotypic data, we are able to explicitly normalize the POE values in metastatic tumors to those in localized tumors. This involves making the likelihood a classification likelihood in which we know the identities of the samples. Letting $G_{L}$ and $G_{M}$ denote sets of sample labels for the localized and metastatic tumor groups, respectively, we can write the likelihood as

$$\begin{array}{lll} ℒ(\mu_{i},\sigma_{i}^{2}) & = & \prod_{i\in G_{L}}N(x_{ij}|\mu_{i},\sigma_{i}^{2})\\ & \times & \prod_{j\in G_{M}}{\left(U(x_{ij}|a_{i},b_{i})\right)}^{e_{ij}}{\left(N(x_{ij}|\mu_{i},\sigma_{i}^{2})\right)}^{1-e_{ij}} \end{array}$$

The derivation of the E- and M steps remains the same except that at the M step,

$$\pi_{i}^{\left(t+1\right)}=\frac{\sum_{j\in G_{M}}e_{ij}^{\left(t+1\right)}}{\left|G_{M}\right|},$$

where |*A| *denotes the cardinality of the set *A*.

The resulting POE can be easily calculated using the simple estimate *P*($G_{M}$) = |$G_{M}$|/*M*_*k*_, where *M*_*k *_is the number of samples in the *k*th study. We plug this estimate into the calculation of POE for all genes and samples. However, at the end of the iteration, we use Bayes' rule to get the posterior probability of *e*_*ij *_being one so that the POE values are not zero.

To see what effects this procedure has, let us consider two hypothetical situations. For the first situation, if the distribution of expression in localized samples overlaps with that of metastatic samples, then most POE values will be near zero. This implies that not many samples will show differential expression between the two groups. By contrast, if the expression values in metastatic samples are concentrated in either tail relative to the distribution of expression in localized samples, then resulting POE values will be larger in absolute value. POE values referenced to one group may reveal the contrast between the two groups, and will help denoise relative expression differences in the two groups when merging across independent datasets, which is otherwise potentially susceptible to other grouping or study-specific effects. Such an adjustment can also be easily incorporated in the full Bayesian model. In our meta-analysis example, the basic principle for both estimation procedures is to fix *e*_*ij *_= 0 for localized tumor samples across all genes throughout, and estimate mixture proportion parameters *π*. With the *π *thus obtained, we can estimate POE for both localized and metastatic tumors. The Bayesian algorithm fixes *e*_*ij *_= 0 for the localized tumors during each iteration of the Gibbs sampling algorithm.

### Interpretation of Latent Variables

Note that the *e*_*ij *_are the target quantities of interest within each study. In particular, we note that much of the meta-analysis literature for genomic data has focused on using gene-specific summaries and combining them across studies (e.g., [3,6]). We instead transform the raw data directly; this may lead to potential gains in efficiency of analysis.

While we describe potential meta-analytic approaches in the next section, we mention two interpretations of the *e*_*ij *_themselves. First, we can define sample subtypes based on the *e*_*ij*_; this was also noted in [32]. Recently, the authors of [33] proposed an alternative to the t-test for biomarker discovery. In this approach, the idea was that a fraction of the cancer samples would be positive for the biomarker(s) of interest so that a t-test would fail to capture the difference. The mixture models described in this paper provide model-based alternatives to the nonparametric testing procedure proposed in [33]. A second interpretation of *e*_*ij *_pertains to the false discovery rate [9]. The false discovery rate is roughly defined to be the expected number of false discoveries divided by the number of rejected hypotheses, where the gene-specific null hypothesis is that there is no differential expression for the gene. It can be shown that for the mixture models in this paper, *P*(*e*_*ij *_= *0|X*) represents the Bayesian false discovery rate [34].

### Integration of Transformed Data: Analysis Tools

Let *X*_*k *_be the study-wise transformed expression data for the study *k*. After fitting one of the two types of models described previously, the data we have are $X_{k}^{*}\equiv P_{k}^{d}$, where $P_{k}^{d}$ is a probability matrix with entries $p_{ij}^{*}\equiv p_{ij}^{+}-p_{ij}^{-}$ as described earlier. For a common set of *N *genes that are profiled in each of the studies of interest, data integration is subsequently based on the rescaled values $X_{k}^{*}$, and results in a combined data matrix of dimension $N\times \sum_{k=1}^{K}M_{k}$. These are the estimated POE values obtained from fitting one of the two algorithms to each of the separate datasets.

Treating the $X_{k}^{*}$ as the new "data," we can now apply standard microarray methodologies to them. Note that one major assumption made is that the studies being combined show relatively little between-study heterogeneity. This is a crucial assumption in meta-analysis [1] and is not any different for the approach we describe here. If this assumption fails, then in effect we would be attempting to combine quantities representing fundamentally different things.

One goal in genomic data analyses is determining which genes are differentially expressed. There are many available methods for doing this, such as SAM [29], analysis using q-values [4] or Empirical Bayes methods [35]. These methods provide adjusted t-statistics for comparing two groups. The adjustment is for the multiple comparisons problem; we prefer approaches based on the false discovery rate because they tend to be less conservative and can provide investigators with calibrated lists of genes that they can then attempt to validate. In the example discussed in the **Results **section, the group label is the tumor type (localized/metastatic).

Another important goal of genomic data analysis is to determine if there exists a gene expression profile that can distinguish the two groups. This is referred to as classification and is widely performed in gene expression studies. We deal with the two-group classification problem here. We use the compound covariate predictor [36] here; other classification procedures could also be used. The compound covariate predictor method works as follows:

(a) Fit a univariate logistic regression model using group label as the response and gene expression as the predictor; obtain the estimated regression coefficient.

(b) Define a risk index as the weighted average of gene expression, where the weight is the estimated regression coefficient from step (a).

We will assess the performance of the genes found using classification accuracy. If we want to assess the performance of the classifier, we must deal with the issue of training and testing the model using the same data. An honest estimate of the prediction error rate is obtained using leave-one-out cross-validation. Define the risk index for sample *j *by $RI_{j}=\sum_{i=1}^{p}{\hat{\beta }}_{i,-j}x_{ij}^{*}$, where $x_{ij}^{*}$ is the POE value for gene *i *in sample *j*, *j *= 1, ..., $\sum_{k=1}^{K}M_{k}$ and ${\hat{\beta }}_{i,-j}$ is the effect estimate for gene *i *in the combined dataset without the sample *j*. The risk index for sample *j *is a weighted linear combination of the expression profiles of the top *p *genes, where the ranking of the genes is based on their corresponding significance in the univariate logistic model fit. As a result, large positive values of *RI *indicate high risk of failure, whereas large negative values of *RI *indicate low risk of failure. Classification of sample *j *to the risk groups is then based on the *j*^th ^leave-one-out risk index. The classifier is $C_{j}$(X*) = *I*{*RI*_*j *_> *c*}, with *c *being the empirical quantiles of the *RI's*. The number of genes *p *in a classifier is also treated as a parameter and optimized to minimize the prediction error rates. Note that to maintain unbiasedness of the procedure, we re-fit the univariate logistic regressions for each dataset with a withheld sample and re-rank genes based on the corresponding results. One other issue involves the use of POE values on training versus testing sets. As described in the previous section, we can obtain POE values either using or not using the class labels. In training sets, we typically use the class labels. We cannot use them in the test set as this will lead to overfitting. However, it is still possible to get POE values without using the phenotypic information for the test set.

## Competing interests

The author(s) declares that there are no competing interests.

## Authors' contributions

DG and HC conceived the method and prepared the manuscript. HC implemented the software and performed the analyses. RS and AC contributed to the discussion. All authors have read and approved the final manuscript.

## Supplementary Material

Additional file 1 Dendrogram of samples from liver data using the Conlon signature. This is a heatmap representing the hierarchical clustering results of the data in [12] using the genes selected by the method of [13]. Click here for file

Additional file 2 Dendrogram of samples from lung data using the Conlon signature. This is a heatmap representing the hierarchical clustering results of the data in [11] using the genes selected by the method of [13]. Click here for file

Additional file 3 Dendrogram of samples from prostate data using the Conlon signature. This is a heatmap representing the hierarchical clustering results of the data in [10] using the genes selected by the method of [13]. Click here for file

Additional file 4 Derivation of conditional distributions for the MCMC-based POE algorithm. This file contains the details of the full conditional distributions from which samples of posterior distribution are drawn. Click here for file
