# Supplementary material for: A Latent Variable Approach for Meta-Analysis of Gene Expression Data from Multiple Microarray Experiments
Source: BMC Bioinformatics. 2007 Sep 27;8:364. doi: 10.1186/1471-2105-8-364 (PMC2246152; doi:10.1186/1471-2105-8-364)
Supplement: Additional file 1 — Dendrogram of samples from liver data using the Conlon signature. This is a heatmap representing the hierarchical clustering results of the data in [12] using the genes selected by the method of [13]. [file 1471-2105-8-364-S1.pdf]

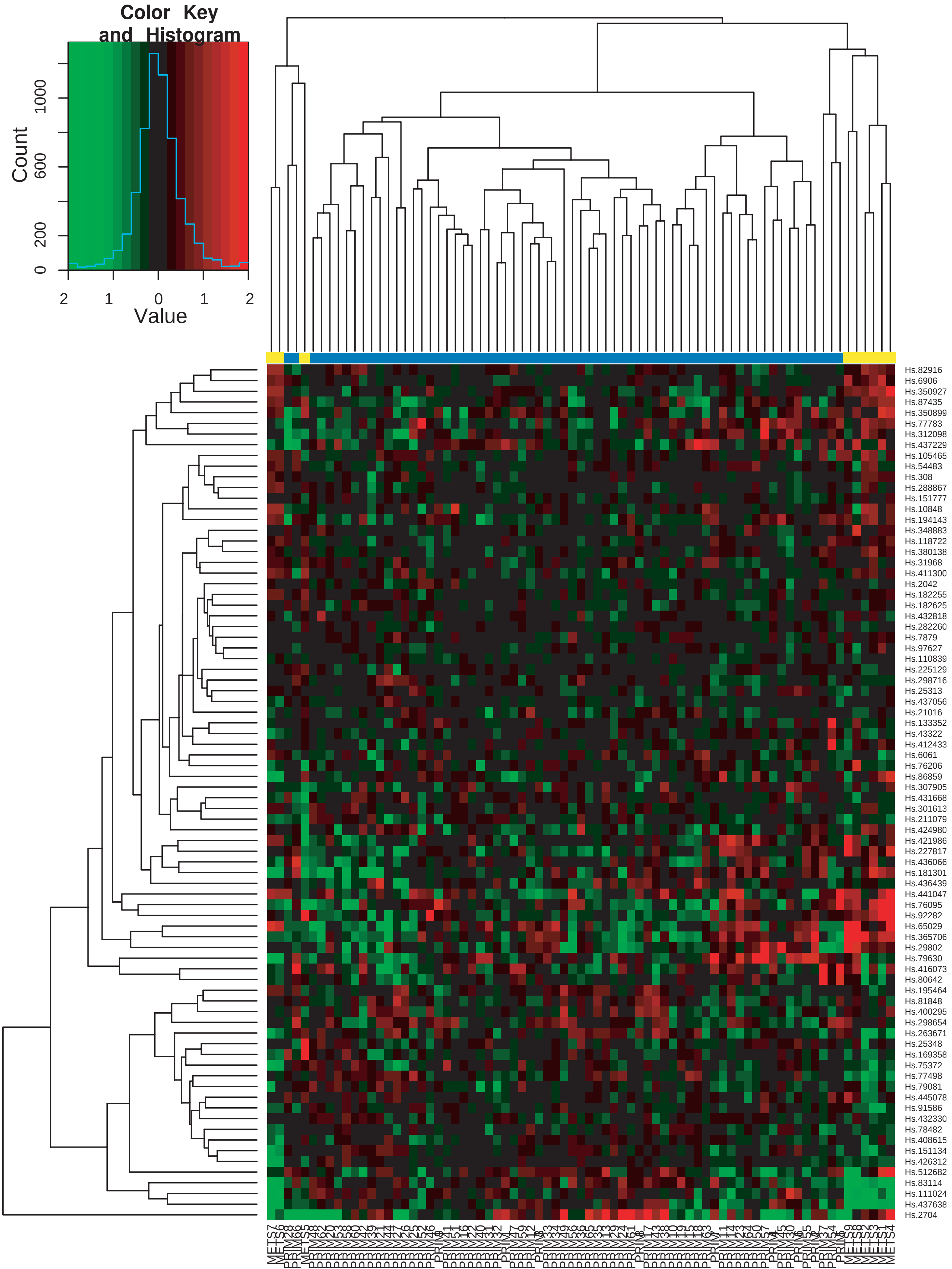

Color Key
and Histogram
Count
0
200
600
1000
2
1
0
1
2
Value
Hs.82916
Hs.6906
Hs.350927
Hs.87435
Hs.350899
Hs.77783
Hs.312098
Hs.437229
Hs.105465
Hs.54483
Hs.308
Hs.288867
Hs.151777
Hs.10848
Hs.194143
Hs.348883
Hs.118722
Hs.380138
Hs.31968
Hs.411300
Hs.2042
Hs.182255
Hs.182625
Hs.432818
Hs.282260
Hs.7879
Hs.97627
Hs.110839
Hs.225129
Hs.298716
Hs.25313
Hs.437056
Hs.21016
Hs.133352
Hs.43322
Hs.412433
Hs.6061
Hs.76206
Hs.86859
Hs.307905
Hs.431668
Hs.301613
Hs.211079
Hs.424980
Hs.421986
Hs.227817
Hs.436066
Hs.181301
Hs.436439
Hs.441047
Hs.76095
Hs.92282
Hs.65029
Hs.365706
Hs.29802
Hs.79630
Hs.416073
Hs.80642
Hs.195464
Hs.81848
Hs.400295
Hs.298654
Hs.263671
Hs.25348
Hs.169358
Hs.75372
Hs.77498
Hs.79081
Hs.445078
Hs.91586
Hs.432330
Hs.78482
Hs.408615
Hs.151134
Hs.426312
Hs.512682
Hs.83114
Hs.111024
Hs.437638
Hs.2704
