# Supplementary material for: A Latent Variable Approach for Meta-Analysis of Gene Expression Data from Multiple Microarray Experiments
Source: BMC Bioinformatics. 2007 Sep 27;8:364. doi: 10.1186/1471-2105-8-364 (PMC2246152; doi:10.1186/1471-2105-8-364)
Supplement: Additional file 4 — Derivation of conditional distributions for the MCMC-based POE algorithm. This file contains the details of the full conditional distributions from which samples of posterior distribution are drawn. [file 1471-2105-8-364-S4.pdf]

Appendix A. Details of Markov Chain Monte Carlo

For interested readers, we explain the approach of Parmigiani et al. (2002) in more detail, particularly the sampling procedures of the actual implementation of Gibbs sampler there, and its subsequent implementation in the metaArray package. We again suppress dependence on the study indicator k. In the algorithm, the gene-specific parameters were repeatedly drawn from the full conditional distributions as shown below. For some parameters, such as κ's, practical considerations lead to a sampling scheme slightly different from the analytically derived conditional distributions.

Define $a \wedge b$ and $a \vee b$ to be the minimum and maximum of a and b. Letting Θ denote the set of all unspecified parameters for each case and $x^{\star}_{ij} = x_{ij} - \mu_i^{(t-1)} - \alpha_j^{(t-1)}$, we first sample boundaries of uniform components:

$$\begin{aligned}\kappa_i^{+(t)}|X, \Theta^{(t-1)} &\sim \max\big((x^{\star}_{i1} \vee 0), \ldots, (x^{\star}_{iM_k} \vee 0)\big) + \mathcal{E}(\theta_\kappa^{+(t-1)}) \\ \kappa_i^{-(t)}|X, \Theta^{(t-1)} &\sim \min\big((x^{\star}_{i1} \wedge 0), \ldots, (x^{\star}_{iM_k} \wedge 0)\big) + \mathcal{E}(\theta_\kappa^{-(t-1)})\end{aligned}$$

where $\mathcal{E}$ denotes exponential distribution and $(\theta_\kappa^{+(t-1)}, \theta_\kappa^{-(t-1)})$ are the rate of the distributions on positive and negetive components of the mixture. Upon obtaining κ's, we can calculate $p_{ij}^{+(t)}$ and $p_{ij}^{-(t)}$ with the current estimates of π, α, μ, and κ, and thus sample unobserved latent variables e_{ij} to construct the the matrix $E^{(t)}$. Given e_{ij}, we first draw sample specific effects $\{\alpha_j\}_{j=1}^{M_k}$ from

$$\alpha_j^{(t)}|X, E^{(t)}, \Theta^{(t-1)} \sim \mathcal{N}(m_{\alpha_j}, v_{\alpha_j})$$

where the posterior mean is the usual conjugate weighted average of specified prior mean (zero here) and $\sum_{i=1}^N 1(e_{ij}^{(t)} = 0) \times N^{-1} \sum_{i=1}^N (x_{ij} - \mu_i^{(t-1)})$, and the posterior variance also is weighted average of reciprocals of prior variance (100 here) and observed deviations. Since α's are normal variates subject to zero sum constraint, it can be shown that centering $\{\alpha_j - \bar{\alpha}\}_{j=1}^{M_k}$ ensures that the adjusted samples are indeed from the target multivariate normal distribution in $\mathbb{R}^{M_k}$ under the constraint that puts all the samples in a hyperplane in $\mathbb{R}^{M_k-1}$. This is evident upon considering the joing distribution of $(\alpha_1, \ldots, \alpha_{M_k}, \sum_{j=1}^{M_k} \alpha_j)$ and its Jacobian for centered α's $(\alpha_1 - \bar{\alpha}, \ldots, \alpha_{M_k} - \bar{\alpha})$.

Next comes the sampling of gene specific effects μ_i in the same fashion using conjugacy of normal distribution, with extra step of sampling σ_i^2 along with μ_i. It is more convenient to introduce some notation here. Set $r_i^{(t)} =$

$\sum_{j=1}^{M_k}(1-e_{ij}^{+(t)}-e_{ij}^{-(t)})$, $\bar{x}_i^{(t)} = \sum_{j=1}^{M_k}(1-e_{ij}^{+(t)}-e_{ij}^{-(t)})x_{ij}/r_i^{(t)}$, and $s_i^{2(t)} = \sum_{i=1}^{M_k}(1-e_{ij}^{+(t)}-e_{ij}^{-(t)})(x_{ij}-\mu_i^{(t-1)}-\alpha_j^{(t)})^2$. Then sample σ^2 and μ as follows, under the assumption that

$$\sigma_i^{-2(t)}|X, E^{(t)}, \alpha^{(t)}, \Theta^{(t-1)} \sim \mathcal{G}\left(\gamma + \frac{r_i^{(t)}}{2}, \lambda + \frac{s_i^{2(t)}}{2}\right)$$

$$\mu_i^{(t)}|X, E^{(t)}, \sigma_j^{-2(t)}, \alpha^{(t)}, \Theta^{(t-1)} \sim \mathcal{N}\left(\frac{r_j^{(t)}\bar{x}_j^{(t)}/\sigma_j^{2(t)} + \xi/\tau^2}{r_j^{(t)}/\sigma_j^{2(t)} + 1/\tau^2}, (r^{(t)}/\sigma_j^{2(t)} + 1/\tau^2)^{-1}\right)$$

which may differ from one's analytical derivation of the posterior. (ξ, τ^2) are the prior mean and variance of μ, (γ, λ) are the shape and scale parameters of gamma distribution of σ^2 there.

The mixture proportions (π^+, π^-) can be drawn by rejection sampling, with proposal from the beta distribution. Acceptance probability can be obtained using the fact that $logit(\pi^+)$ and $logit(\pi^-)$ are assumed to be normally distributed. The proposal of new values is through:

$$\begin{aligned}
\pi_i^{+(t)}|X, E^{(t)}, \Theta^{(t)} &\sim \mathcal{B}(1 + \sum_{j=1}^{M_k} 1(e_{ij}^{(t)} = 1), 1 + M_k - \sum_{j=1}^{M_k} 1(e_{ij}^{(t)} = 1)) \\
\pi_i^{-(t)}|X, E^{(t)}, \Theta^{(t)} &\sim \mathcal{B}(1 + \sum_{j=1}^{M_k} 1(e_{ij}^{(t)} = -1), 1 + M_k - \sum_{j=1}^{M_k} 1(e_{ij}^{(t)} = -1))
\end{aligned}$$

where $\mathcal{B}$ is Beta distribution. Equivalently, one can sample directly from Dirichlet distribution. Then the Metropolis Hastings ratio is the ratio of normal density evaluated at the proposal samples and samples at previous stage, giving acceptance probability. Having sampled all relevant parameters, we can now calculate

$$p_{ij}^{+(t)} = \frac{\pi_i^{+(t)} f_1(x_{ij}; \mu_i^{(t)}, \kappa_i^{+(t)})}{f(x_{ij}; \Theta_i^{(t)})} \qquad p_{ij}^{-(t)} = \frac{\pi_i^{-(t)} f_{-1}(x_{ij}; \mu_i^{(t)}, \kappa_i^{-(t)})}{f(x_{ij}; \Theta_i^{(t)})},$$

which forms an approximate sample from posterior distribution of $p_{ij}^{(t)} = p_{ij}^{+(t)} - p^{-(t)}$. The derivation of these conditionals is fairly standard; see Diebolt and Robert (1994). To use the phenotypic information, set $e_{ij} = 0$ for the reference group (e.g. localized tumors in our meta-analysis example) and unknown for the remaining samples.
